# Intertemporal decision-making as a mediator between personality traits and self-management in type 2 diabetes: a cross-sectional study

**DOI:** 10.3389/fpsyg.2023.1210691

**Published:** 2023-07-28

**Authors:** Linfang Deng, Shaoting Luo, Qianna Fang, Jinjiang Xu

**Affiliations:** ^1^Department of Nursing, Jinzhou Medical University, Jinzhou, China; ^2^Department of Pediatric Orthopedics, Shengjing Hospital of China Medical University, Shenyang, China; ^3^Department of Health Management Center, The First Hospital of Jinzhou Medical University, Jinzhou, China

**Keywords:** self-management, intertemporal decision-making, type 2 diabetes mellitus, personality traits, mediation analysis

## Abstract

**Objectives:**

The aims to investigate the mediating effect of intertemporal decision-making on the association between personality traits and self-management among individuals with in Type 2 Diabetes (T2DM).

**Method:**

Patients with T2DM in the early stages of hospitalization at two tertiary hospitals in Shenyang and Jinzhou, Liaoning Province, May 2022 to January 2023. Questionnaires, including General Demographic, Self-Management, Big Five Personality, and Intertemporal Decision-Making, were administered. Pearson correlation analysis examined relationships between personality traits, intertemporal decision-making, and self-management. Hierarchical regression analysis identified self-management predictors. Mediation analysis used the PROCESS SPSS Macro version 3.3 model 4 to investigate intertemporal decision-making as mediator between personality traits and self-management.

**Results:**

Pearson correlation analysis revealed significant associations between self-management scores, personality traits, and intertemporal decision-making. Hierarchical regression revealed that Neuroticism and Conscientiousness accounted for 20.8% of the variance in self-management, while intertemporal decision-making explained 4.5% of the variance. Finally, using the Bootstrap method, the mediation analysis showed that intertemporal decision-making partially mediated the effect of personality traits on self-management.

**Conclusion:**

This study emphasizes the importance of intertemporal decision-making in improving self-management behaviors among patients with T2DM. Interventions targeted at modifying intertemporal decision-making preferences could be effective in enhancing self-management behaviors, leading to better health outcomes.

## 1. Introduction

Type 2 diabetes mellitus (T2DM) is a prevalent non-communicable disease globally and poses a significant public health challenge (World Health Organization, 2016a). According to the International Diabetes Federation (IDF), approximately 451 million individuals worldwide are affected by diabetes, and this number is projected to rise to 693 million by 2045 (Cho et al., 2018). Notably, China has the highest number of individuals with diabetes globally, representing 24% of the global diabetic population (Li Y. et al., 2020).

According to data from the World Health Organization (WHO), diabetes results in an estimated 60,000 fatalities annually (World Health Organization, 2016b). Achieving adequate glycemic control in patients with T2DM is critical in reducing morbidity and mortality from diabetic complications [UK Prospective Diabetes Study (UKPDS) Group, 1998]. This highlights the crucial role of self-management in patients with T2DM. In other words, effective self-management is essential in achieving optimal glycemic control and reducing the risk of complications among patients diagnosed with T2DM. Self-management in patients with T2DM involves a series of behaviors that patients perform to manage their diabetes independently, such as exercise, blood glucose monitoring, diet planning, medication administration, and foot care. However, some studies have indicated that T2DM patients do not adhere well to their prescribed self-management regimen (Yusuff et al., 2008; Wabe et al., 2011). More than three-quarters of these patients do not monitor their blood glucose levels regularly (Adisa and Fakeye, 2014). However, in emphasizing the necessity of improving self-management in patients with Type 2 Diabetes Mellitus (T2DM), we cannot solely focus on the physical complications. Equally important is our recognition of the psychological impacts induced by diabetes, particularly disease-related mental health issues such as anxiety and depression (Amiri and Behnezhad, 2019; Yasui-Furukori et al., 2019; Kang, 2022). The mental health status of diabetic patients is a key factor in their treatment and management (Gonzalez et al., 2008; Kostev and Jacob, 2018). On the other hand, research has also found a negative correlation between diabetes and the scores of Self-Rated Health (SRH), a subjective evaluation of one’s own health status that has profound implications for patient quality of life and disease management (Huang and Garcia, 2019; Kang and Malvaso, 2023). Diabetic patients often begin to perceive a deterioration in their health status at a relatively young age, resulting in lower SRH scores compared to their peers. This may reflect, to some extent, the broad impact of diabetes on patient quality of life, thereby underscoring the urgency of improving self-management in diabetic patients.

With the advent of the physiological-psychosocial model of medicine, psychological factors have garnered significant attention from researchers. Personality refers to the distinct expression of an individual’s emotions, cognition, and behavior, which includes the underlying theoretical mechanisms and features. This psychological construct is relatively stable and typically shaped by both genetic and environmental factors. Personality traits represent a multidimensional psychological construct, reflecting enduring and stable differences in an individual’s behavior, thought, and emotion. These traits form unique patterns of behavior and emotional responses in individuals, thus playing a key role in understanding and predicting human behavior. The Big Five personality trait model, one of the significant models in personality research, consists of five dimensions: Extraversion, Neuroticism, Conscientiousness, Agreeableness, and Openness (Boyle et al., 2008). Previous studies have indicated that personality traits are important predictors of self-management behaviors in patients with T2DM (Lane et al., 2000; Skinner et al., 2014; Dadras et al., 2022). This perspective has also been validated in research concerning patients with glaucoma (Chen, 2019), cancer chemotherapy (Magalhães et al., 2020), and bipolar affective disorder (Vierck and Joyce, 2015). Nevertheless, more in-depth studies are needed in order to determine which specific personality traits are associated with self-management behaviors in patients with T2DM. Furthermore, the association between different personality traits and patient self-management remains controversial. For instance, Adachi et al. (2022) reported a significant positive correlation between conscientiousness and medication adherence in patients with cardiovascular diseases, with no significant relationship observed with other personality traits. Similarly, Abu et al. (2023) found that both conscientiousness and agreeableness could significantly predict medication adherence, while neuroticism, extraversion, and openness to experience demonstrated no significant association.

Therefore, it is vital to examine the self-management behaviors of patients with type 2 diabetes from the perspective of personality traits.

According to the health belief model, individuals’ beliefs about their health and susceptibility to disease impact their decision-making behavior. Intertemporal decision-making refers to individuals’ choices about trade-offs between different periods, particularly the present and future, especially when presented with smaller immediate rewards versus larger delayed rewards (Lempert and Phelps, 2016). For patients with diabetes, a preference for immediate gratification may result in short-term behaviors such as consuming high-sugar or high-fat foods, which can impact self-management. Studies have established a correlation between intertemporal decision-making and self-management in patients with T2DM (Karl et al., 2018; Madsen et al., 2019). Additionally, personality traits can predict patients’ behavioral tendencies when weighing immediate versus delayed consequences of health and exercise behaviors. Research indicates that patients with T2DM with high neuroticism may be more inclined toward immediate gratification, such as opting out of exercise or choosing sugary foods, as they may find it more difficult to endure temporary discomfort. In contrast, patients high in conscientiousness might be more oriented toward long-term health outcomes, willing to forgo immediate pleasure, such as maintaining regular exercise and healthy eating habits, in pursuit of long-term wellness (Li Z. M. et al., 2020).Thus, we hypothesize that intertemporal decision-making may mediate the relationship between personality traits and self-management in patients with T2DM.

In this study, we used the health belief model as a theoretical framework to examine the impact of intertemporal decision-making and personality traits on self-management, as well as the mediating effect of intertemporal decision-making between personality traits and self-management. The aim is to provide healthcare professionals with a theoretical basis for implementing effective interventions to enhance self-management in this patient population.

## 2. Methods

### 2.1. Participants

In this study, a convenience sampling method was used to recruit 240 patients with T2DM in the early stages of hospitalization at two tertiary hospitals in Shenyang and Jinzhou, Liaoning Province, between May 2022 and January 2023. The inclusion criteria were: (1) Confirmed diagnosis of type 2 diabetes according to WHO’s diagnostic criteria (World Health Organization, 2011); (2) Admission within 1 day; (3) Age of 18 years or older; (4) Signed informed consent form. The exclusion criteria were: (1) Serious illnesses such as malignancy, heart disease, liver disease, etc.; (2) Acute onset conditions such as diabetic ketoacidosis; (3) Mental illness: Patients assessed by a psychiatrist and found to conform to the DSM-5 (Diagnostic and Statistical Manual of Mental Disorders, 5th Edition) criteria for mental disorders.

This study is a cross-sectional survey study, and the sample size was determined based on the overall mean. Using the formula *n* = (uɑ/2σ/δ)2, with a significance level of *ɑ* = 0.05 and uɑ/2 = 1.96, The value of *σ* = 35.38 was obtained from a review of the literature, and a tolerance error of *δ* = 5 was set. The resulting sample size was *n* = (1.96 × 35.38/5)2 = 192. To account for a potential 10% sample loss rate, the final sample size was increased to 240 cases. Each interview lasted about 20 min.

### 2.2. Ethics approval and consent to participate

This study is based on a research project approved by the Ethics Committee of Jinzhou Medical University of Medical Sciences with the code of ethics JZMULL2022100. All procedures performed in this study were in accordance with the ethical standards of the institutional and/or national research committee, as well as the 1964 Helsinki Declaration and its later amendments, or their equivalent. Written informed consent was obtained from all participants.

### 2.3. Measures

1. General information: including age, gender, place of residence, occupation, Education level, monthly family income, duration of disease, family history of diabetes, and medication regimen of T2DM patients.
2. Intertemporal decision-making: The intertemporal decision-making questionnaire developed by Chen (Chen and He, 2011) et al. in their experimental study was widely used in related studies in China. The questionnaire had 19 items, with two options for each item: one of which was an immediate option now, and the other was a delayed option 6 months later. The initial value of the immediate option reward was 50 RMB and increased in increments of 50 RMB, while the reward for the 6-month later option was always 1,000 RMB. The scoring rules state that the participant considers the immediate option as the turning point of the intertemporal decision-making process when they first select it. The subjective value of the delayed option is calculated as the average of that option and the previous immediate option. A smaller subjective value indicates a more “short-sighted” participant who opts for the current option earlier.
3. Personality characteristics of patients with T2DM: A short version of the Chinese Big Five Personality Questionnaire, developed by Wang et al. (2011). This questionnaire consisted of 40 entries in 5 dimensions, including Extraversion, Conscientiousness, Agreeableness, Neuroticism, and Openness, each responding to different personality traits. Patients rated their responses on a Likert 6-point scale ranging from “very nonconforming = 1 to very conforming = 6.” Higher scores in each dimension indicated more pronounced traits in that dimension. The Cronbach’s coefficient for each dimension ranged from 0.529 to 0.770 using the Likert 6-point scale, and the Cronbach’s coefficient for the total scale was 0.793.
4. Self-management of T2DM patients: The Self-Management Behavior Scale for Diabetic Patients, developed by foreign scholar Toobert et al. (2000) and translated by Chinese scholar Wan et al. (2008), was used. The scale consisted of six dimensions: diet, exercise, blood glucose monitoring, foot care, and medication, with higher scores indicating stronger individual self-management. The scale demonstrated good reliability and validity with a Cronbach’s coefficient of 0.62 and a retest reliability of 0.83.

### 2.4. Analysis

The data were analyzed using the IBM SPSS Statistics software package, version 21.0 for Windows. Descriptive statistics such as means, standard deviations, frequencies, and percentages were used to describe the basic characteristics of patients with type 2 diabetes mellitus (T2DM). Two independent samples t-test or analysis of variance (ANOVA) were used to analyze the differences in demographic data for self-management. Pearson correlation analysis was used to examine the relationship between self-management, personality traits, and intertemporal decision-making. Hierarchical regression analysis was used to determine significant predictors of self-management. The PROCESS program in SPSS software was used to test the mediating effect of intertemporal decision-making in the relationship between personality traits and self-management based on Bootstrap 5,000 self-sampling.

## 3. Results

### 3.1. Characteristics of the study participants (*n* = 240)

In this study, 240 patients diagnosed with type 2 diabetes mellitus (T2DM) were investigated (Table 1). Of these patients, 59.2% were female, and 98.3% were married. The majority of patients were aged between 50 and 60 years, accounting for 40.8% of the total. Junior high school was the predominant education level, with 83 cases accounting for 34.6%. Farmers were the most common occupation, accounting for 106 cases or 44.2%. Most patients had a monthly family income between ¥2000–4000, accounting for 38.8%. The majority of patients (78.3%) lived with their spouses, and 75.8% used urban residents’ medical insurance as their payment method. Patients with disease duration of 10 years or more accounted for 39.6% of the total. The medication regimen was dominated by patients using insulin and oral medication, accounting for 38.8%. A further comparison of self-management scores of type 2 diabetes patients with different demographic characteristics showed significant differences (*p* < 0.05) between self-management and age, gender, education level, monthly household income, disease duration, family history of diabetes, and occupation. Patients aged over 60 years had significantly higher self-management scores than patients in other age groups (*p* < 0.001). Women had significantly higher self-management scores than men (*p* < 0.001). Patients with higher levels of education showed higher scores of self-management behaviors (*p* < 0.001). Patients with type 2 diabetes with a disease duration of 6–9 years had the highest self-management scores, significantly higher than those with other disease durations (*p* < 0.001). Patients with a family history of diabetes had higher self-management scores than those without a family history of diabetes (*p* < 0.05), and the difference was statistically significant. In addition, we found that gender had a moderate effect on self-management scores (Cohen’s *d* = 0.595, *p* < 0.001), women scored significantly higher than men. Similarly, having a family history of diabetes also affected self-management scores, although the effect size was small (Cohen’s *d* = 0.294, *p* < 0.05).The results are shown in Supplementary Table 1.

**Table 1:** Difference in self-management in demographic factors of patients with T2DM.

Characteristics	*N*(%)	Self-Management		
		Mean(SD)	*t*/*F*	*p*-value
Age				
<40	23(9.6%)	27.83(7.171)	2.616^b^	0.001^*^
40-	43(17.9%)	32.09(8.986)		
50-	98(40.8%)	38.32(6.844)		
60-	76(31.7%)	40.79(8.253)		
Gender				
Male	98(40.8%)	33.97(9.166)	−4.596^a^	0.001^*^
Female	142(59.2%)	39.06(7.881)		
Living conditions				
Living alone	3(1.3%)	35.33(4.933)	0.805^b^	0.794
Living with spouse	188(78.3%)	36.78(9.124)		
Living with parents	4(1.7%)	32.00(7.348)		
Living with children	45(18.8%)	38.38(7.423)		
Education level				
Primary and below	60(25.0%)	34.83(8.087)	2.044^b^	0.001^*^
Junior high school	83(34.6%)	36.60(6.884)		
High/Junior College	58(24.2%)	38.29(9.353)		
University/college	39(16.3%)	39.13(11.594)		
Marital status				
Unmarried	4(1.7%)	31.00(5.598)	−1.377^a^	0.170
Marriage	236(98.3%)	37.08(8.793)		
Monthly household income				
≤2000	92(38.3%)	33.46(7.947)	2.975^b^	0.001^*^
2000–4000	93(38.8%)	36.77(7.346)		
≥4000	55(22.9%)	43.22(9.036)		
Disease duration				
≤1 years	8(3.3%)	26.75(6.341)	3.018^b^	0.001^*^
1–5 years	60(25.0%)	30.92(8.189)		
6–9 years	77(32.1%)	33.99(8.268)		
≥10 years	95(39.6%)	40.85(6.863)		
Medication regimen				
Diet exercise	1(0.4%)	13.00(0.001)	1.377^b^	0.079
Oral medication only	61(25.4%)	37.51(5.912)		
Insulin only	85(35.4%)	35.49(10.120)		
Insulin + oral medication	93(38.8%)	38.25(8,583)		
Ethnicity				
Ethnic Han	223(92.9%)	37.02(8.722)	0.247^a^	0.805
Others	17(7.1)	36.47(9.722)		
Place of residence				
Rural	120(50%)	37.70(8.344)	1.274^a^	0.204
Urban	120(50)	36.26(9.166)		
Occupation				
Worker	14(5.8%)	32.43(6.111)	5.366^b^	0.001^*^
Farmer	106(44.2%)	35.87(7.810)		
Retire	71(29.6%)	38.72(8.571)		
Administrative cadre	19(7.9%)	31.11(7.164)		
Teacher	4(1.7%)	34.00(10.100)		
Professionals	15(6.3%)	44.40(10.568)		
Individual merchant	11(4.6%)	43.36(9.902)		
Family history of diabetes				
Yes	98(40.8%)	38.04(8.176)	2.263^a^	0.025^*^
No	142(59.2%)	35.45(9.413)		
Diabetes education				
Yes	184(76.7%)	36.90(8.741)	0.246^a^	0.806
No	56(23.3%)	37.23(8.965)		
Medical insurance				
Residents’ medical	182(75.8%)	37.06(8.375)	0.254^a^	0.800
Employees’ medical	58(24.2%)	36.72(10.007)		

SD, standard deviation.

a *t*-test.

b *F*-test.

* *p* < 0.05.

### 3.2. The self-management, personality traits, and intertemporal decision-making scores of T2DM patients (*X* ± *S*, *n* = 240)

The total self-management score for T2DM patients was (36.98 ± 8.776). The scores for the five dimensions of personality traits were as follows: Extraversion (27.36 ± 6.396), Conscientiousness (32.62 ± 5.546), Neuroticism (27.53 ± 6.817), Agreeableness (33.09 ± 4.825), and Openness (28.72 ± 6.844). The intertemporal decision-making score was (224.380 ± 169.202). Other dimensional scores for patients with T2DM are shown in Table 2.

**Table 2:** The self-management, personality traits, and Intertemporal decision-making scores of T2DM patients (*X* ± *S*, *n* = 240).

Variable	Items	Mean (±SD)	Items mean (±SD)
Self-management	11	36.98 ± 8.776	3.362 ± 0.798
Medication	1	5.60 ± 1.455	5.600 ± 1.455
Exercise	2	5.97 ± 2.307	2.800 ± 1.154
Foot care	2	6.91 ± 3.332	3.455 ± 1.666
Diet	4	13.50 ± 4.708	3.375 ± 1.177
Blood glucose monitoring	2	5.00 ± 2.458	2.500 ± 1.229
Personality traits			
Extraversion	8	27.36 ± 6.396	3.420 ± 0.780
Conscientiousness	8	32.62 ± 5.546	4.078 ± 0.693
Neuroticism	8	27.53 ± 6.817	3.441 ± 0.852
Agreeableness	8	33.09 ± 4.825	4.136 ± 0.603
openness	8	28.72 ± 6.844	3.590 ± 0.856
Intertemporal decision-making	19	224.380 ± 169.202	11.809 ± 8.905

### 3.3. Correlations between self-management, intertemporal decision-making, and personality traits in patients with T2DM

Table 3 displays the results of the correlation analysis examining the relationships among personality traits, intertemporal decision-making, and self-management in patients with T2DM. The results indicate a significant negative correlation between self-management and Neuroticism (*r* = −0.579, *p* < 0.01) and positive correlations with Conscientiousness and Extraversion (*r* = 0.541, p < 0.01) and (*r* = 0.291, *p* < 0.01), respectively. Additionally, intertemporal decision-making showed a significant positive correlation with self-management (*r* = 0.466, *p* < 0.01). Furthermore, intertemporal decision-making exhibited a significant negative correlation with Neuroticism (*r* = −0.195, *p* < 0.01) and a positive correlation with Conscientiousness (*r* = 0.204, *p* < 0.01). For more detailed information, refer to Table 3.

**Table 3:** Correlation analysis of personality traits, intertemporal decision-making, and self-management.

Variable	Personality traits					Intertemporal decision-making	Self-management
	Neuroticism	Conscientiousness	Openness	Agreeableness	Extraversion		
Neuroticism	1						
Conscientiousness	−0.303^**^	1					
openness	−0.248^**^	0.213^**^	1				
Agreeableness	−1.111	1.152^*^	0.079	1			
Extraversion	−0.285^**^	0.220^**^	−0.020	0.119	1		
Intertemporal decision-making	−0.195^**^	0.204^**^	0.074	−0.051	0.077	1	
Self-management	−0.579^**^	0.541^**^	0.064	0.054	0.291^**^	0.466^**^	1

^*^p < 0.05; ^**^p < 0.01.

### 3.4. Hierarchical regression analysis of self-management in patients with T2DM patients

Table 4 displays the results of a stratified regression analysis of self-management in patients with T2DM. The analysis involved three models. Model 1 served as the control variable model, consisting of independent variables such as age, gender, education, monthly household income, disease duration, occupation, and family history. Model 2 added five dimensions of personality traits (extroversion, Neuroticism, Conscientiousness, Agreeableness, and Openness) to Model 1. Finally, Model 3 included intertemporal decision-making in addition to Model 2’s variables, with self-management being the dependent variable for all models. The results indicate that Model 2 exhibited significant changes in *F*-values (*p* < 0.001) after adding the five personality trait dimensions to Model 1. The *R*^2^ value also increased from 0.454 to 0.622, indicating that Neuroticism and Conscientiousness personality traits explained 20.8% of the variance in self-management. Specifically, (*β* = −0.480, *p* < 0.001, 95% CI [−0.544, −0.323]) and (*β* = 0.359, *p* < 0.001, 95% CI [0.227, 0.491]) for Neuroticism and Conscientiousness, respectively. Furthermore, in Model 3, which included intertemporal decision-making, the change in *F*-values was significant (*p* < 0.001), and the *R*^2^ value increased from 0.662 to 0.707. These findings suggest that intertemporal decision-making explained 4.5% of the variance in self-management. Specifically, (*β* = 0.012, *p* < 0.001, 95% CI [0.008, 0.016]). For further details, see Table 4.

**Table 4:** Hierarchical regression analysis of self-management in patients with T2DM.

Variable	Self-management		
	Mode1 1β(95%CI)	Model 2β(95%CI)	Model 3β(95%CI)
Control variable			
Age	2.571^**^ (1.380,3.762)	1.662^*^(0.691,2.632)	1.539^*^(0.633, 2.445)
Gender	2.233^*^(0.017,0.411)	0.551 (−0.938, 2.039)	0.684(−0.705, 2.072)
Education	0.562(−0.533,31.658)	0.856 (−0.026, 1.739)	0.806(−0.017, 1.629)
Household income	4.209^**^(2.807,5.610)	2.522^**^(1.355, 3.689)	2.356^**^(1.266, 3.445)
Disease duration	2.523^**^(1.243,3.803)	1.738^*^(0.709, 2.767)	1.555^*^(0.594, 2.516)
Occupation	−0.131(−0.904,0.642)	0.123 (−0.499, 0.744)	−0.117(−0.702, 0.468)
Family history	−1.015(−2.798,0.769)	−0.308 (−1.737, 1.120)	−0.318(−1.650, 1.014)
Personality traits			
Extraversion		0.083(−0.029, 0.194)	0.085(−0.019, 0.189)
Neuroticism		−0.480^**^(−0.598, −0.363)	−0.434^**^(−0.544, −0.323)
Conscientiousness		0.393^**^(0.253,0 0.534)	0.359^**^(0.227, 0.491)
Agreeableness		−0.081(−0.225, 0.062)	−0.043(−0.177,0.092)
Openness		0.076(−0.033,0 0.186)	0.055(−0.047, 0.158)
Intertemporal decision-making			0.012^**^(0.008, 0.016)
*F*	27.578^**^	37.061^**^	42.031^**^
*R* ^2^	0.454	0.662	0.707
△*R*^2^	0.454	0.208	0.045

^*^p < 0.05; ^**^p < 0.001.

### 3.5. The mediating role of intertemporal decision-making in patients with T2DM

**Figure 1:**
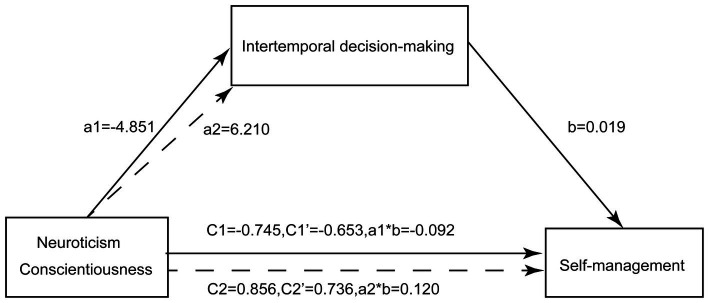
Mediating effects of intertemporal decision-making in the personality traits (Neuroticism-solide line/Conscientiousness-dash line) and self-management. a1: effect of Neuroticism on intertemporal decision-making; a2: effect of Conscientiousness on intertemporal decision-making; b: effect of intertemporal decision-making on self-management; c’: direct effect of Neuroticism/Conscientiousness after adjustment for intertemporal decision-making; a*b: mediating effects of intertemporal decision-making between Neuroticism/ Conscientiousness and self-management.

A mediating effects model was created to examine the relationship between neurotic personality traits and self-management in patients with T2DM. In the model, self-management was the dependent variable, neurotic personality traits were the independent variable, and intertemporal decision-making served as the mediating variable. The model is illustrated in Figure 1. The results of the study indicate that Neuroticism had a significant negative effect on both self-management (*β* = −0.745, *p* < 0.01) and intertemporal decision-making (*β* = −4.851, *p* < 0.01). However, when both intertemporal decision-making and Neuroticism were included in the model simultaneously, the negative effect of Neuroticism on self-management decreased (*β* = −0.653, *p* < 0.01), as shown in Table 5. The indirect effect of 95% CI did not include 0, indicating that the mediating effect of intertemporal decision-making on the relationship between Neuroticism and self-management was significant. This suggests that Neuroticism affects self-management in patients with T2DM by influencing intertemporal decision-making. The direct effect of 95% CI did not include 0, indicating that the effect of intertemporal decision-making on the relationship between Neuroticism and self-management was partially mediated. The mediating effect was −0.092, and it accounted for 12.35% of the total effect. The 95% CI for the mediating effect was (−0.161, −0.028). These findings are presented in Table 6.

**Table 5:** The mediating role of intertemporal decision-making in patients with T2DM.

Variable	Predictive variable	Fitting index		Coefficient significance	
		*R* ^2^	*F*	*t*	*β*
Self-management	Neuroticism	0.335	120.112	−10.960	−0.746^**^
Intertemporal decision-making	Neuroticism	0.038	9.450	−3.074	−4.851^**^
Self-management	Neuroticism	0.465	102.855	−10.472	−0.653^**^
	Intertemporal decision-making			7.565	0.019^**^
Self-management	Conscientiousness	0.292	98.361	9.918	0.856^**^
Intertemporal decision-making	Conscientiousness	0.041	10.284	3.207	6.210^**^
Self-management	Conscientiousness	0.424	87.381	9.243	0.736^**^
	Intertemporal decision-making			7.372	0.019^**^

^**^p<0.01.

**Table 6:** The mediating effects test for intertemporal decision-making in patients with type 2 diabetes.

Effect	Path	*β*	Standard error	95%CI		Relative effect value
				Lower	Upper	
Total effect	Neuroticism → Self-Management	−0.745	0.068	−0.88	−0.612	
Mediating effect	Neuroticism → Intertemporal decision-making	−0.092	0.033	−0.161	−0.028	12.35%
	Intertemporal decision-making → Self management					
Direct effect	Neuroticism → Self-management	−0.653	0.062	−0.779	−0.532	87.65%

Effect		Regression coefficient	Standard error	95%CI		Relative effect value
				Lower	Upper	
Total effect	Conscientiousness → Self-management	0.856	0.086	0.686	1.026	
Mediating effect	Conscientiousness → Intertemporal decision-making	0.120	0.035	0.055	0.195	14.02%
	Intertemporal decision-making → Self-management					
Direct effect	Conscientiousness → Self-management	0.736	0.081	0.583	0.898	85.98%

The study findings indicate that Conscientiousness personality traits had a positive effect on both self-management (*β* = 0.856, *p* < 0.01) and intertemporal decision-making (*β* = 6.210, *p* < 0.01). However, when intertemporal decision-making and Conscientiousness personality traits were entered simultaneously, the positive effect of Conscientiousness on self-management decreased (*β* = −0.736, *p* < 0.01), as shown in Table 5. The indirect effect with a 95% CI excluding 0 indicated that the mediating effect of intertemporal decision-making was significant in explaining the relationship between Conscientiousness personality traits and self-management in patients with type 2 diabetes. This suggests that Conscientiousness personality traits affect self-management by influencing intertemporal decision-making. The direct effect with a 95% CI excluding 0 indicated that the effect of intertemporal decision-making on the relationship between Conscientiousness and self-management was partially mediated, with a mediating effect of 0.120. The mediating effect accounted for 14.02% of the total effect, and the 95% CI was (0.055, 0.195). These findings are presented in detail in Table 6.

## 4. Discussion

This study found three main results. First, Conscientiousness and Neuroticism personality traits were strongly linked with self-management in patients diagnosed with type 2 diabetes. Second, intertemporal decision-making also had a strong correlation with patients’ self-management. Third, intertemporal decision-making played a mediating role between Conscientiousness, Neuroticism, and self-management. These findings underscore the importance of intertemporal decision-making in the self-management of T2DM patients with various personality traits. Therefore, intertemporal decision-making preferences should be taken into consideration when developing interventions to improve self-management.

This study revealed a low level of self-management in patients diagnosed with T2DM patients (3.362 ± 0.798). This result is consistent with a study conducted in an Arab population (Al-Ozairi et al., 2023), which reported a self-management level of (3.400 ± 1.200) in patients with T2DM. The study also found that patients with T2DM exhibited poor management of blood glucose measurements compared to other aspects of self-management. This inconsistency can be attributed to financial barriers faced by patients. Although 95% of the Chinese population has access to basic social health insurance, which includes the new rural cooperative medical scheme, the basic urban residents’ health insurance scheme, and the basic employees’ insurance scheme (Meng et al., 2015). These insurance plans do not cover the cost of glucose monitoring equipment and test strips. Consequently, patients are burdened with significant financial expenses to acquire the necessary diabetes management tools, which may hinder adequate monitoring and management, adversely affecting disease control and prognosis. On the other hand, fear of needles among patients may also contribute to barriers to self-management of blood glucose in patients with T2DM. The study also discovered that women, older individuals, those with a longer duration of the disease, and higher levels of Monthly household income demonstrated higher levels of self-management among patients with T2DM. Firstly, women may be more attentive and patient when it comes to self-management, leading to higher levels of self-management in women compared to men. Secondly, older patients may place more emphasis on health-related issues and have a greater need for self-management. Moreover, patients with a longer duration of the disease may have acquired more experience and knowledge, leading to better performance in self-management. Lastly, higher levels of Monthly household income may provide patients with easier access to health information and resources, which may enable them to self-manage more effectively.

The study revealed a significant correlation between the self-management ability of patients with T2DM and their Conscientiousness and Neuroticism personality traits. Patients with high levels of Conscientiousness and low levels of Neuroticism demonstrated higher self-management scores, which is consistent with previous research (Wheeler et al., 2012; Skinner et al., 2014). This could be attributed to the fact that patients with high Conscientiousness are more organized and disciplined, which enables them to adhere to self-management behaviors. Additionally, they tend to be more proactive in acquiring information related to their disease and forming strong health beliefs, which motivates the implementation of self-management practices (Skinner et al., 2014). A previous longitudinal survey of adolescents with type 1 diabetes found that those with lower levels of Conscientiousness exhibited poorer self-glucose control after 1 year (Rassart et al., 2018). Conversely, patients with a neurotic personality tend to be emotionally unstable, guilt-ridden, sensitive, and doubtful. These traits can lead to increased stress and negative emotions related to disease management, which may affect their self-management ability. A longitudinal cohort study on patients with T2DM found that those with lower Neuroticism scores exhibited better self-management, leading to improved clinical outcomes (Lane et al., 2000). These findings emphasize the need for interventions aimed at improving self-management to consider the impact of cautiousness and Neuroticism personality traits. Such interventions can help patients develop healthy beliefs and overcome adverse emotions that may negatively affect their self-management behaviors, ultimately promoting effective self-management behaviors. The results of this study suggest that intertemporal decision-making preference plays an important role in the self-management of patients with T2DM. Specifically, patients with delayed gratification preference showed better levels of self-management, whereas patients with immediate gratification preference were more inclined to focus on their current lifestyles rather than adopting behavior change measures, such as diet, exercise, and medication. Additionally, a study of hypertensive patients showed that those with an immediate gratification preference had lower medication adherence compared to those with a delayed gratification preference (Krousel-Wood et al., 2022). These results suggest that interventions for T2DM patients should focus on improving their intertemporal decision-making abilities. This could be done by implementing measures to help patients enhance their delayed gratification skills, which in turn may improve their ability to engage in effective self-management.

The Big Five personality traits have a direct influence on self-management in patients with T2DM, and they also indirectly affect it through intertemporal decision-making. In this study, we found that intertemporal decision-making partially mediates the relationship between Neuroticism/Conscientiousness and self-management. This suggests that while personality traits are relatively stable, we can improve self-management behaviors by addressing the intermediate mediator of personality traits affecting self-management and intertemporal decision-making. However, it is worth emphasizing that intertemporal decision making does not fully explain these associations, and there may be other unknown potential factors influencing self-management behavior. There are several other factors that may contribute to these associations, including cognitive and emotional illness perception, and coping strategies. For instance, a study conducted by Kim et al. (2021) highlighted the significant influence of ‘Type D personality’, ‘cognitive illness perception’, ‘emotional illness perception (Depression)’, and ‘coping strategies (Approach coping)’ on self-management behavior. In addition, consideration of environmental stressors may also contribute to these associations. Patients with Type D personality (TDP) exhibit significantly lower self-efficacy and insufficient self-care behavior. These external pressures and internal psychological conditions could play a key role in the management and control of diabetes (Lin et al., 2020). Future research should further explore these potential influencing factors. Patients with high neurotic personality traits may exhibit impatience and impulsivity, which can lead to inconsistent behaviors in terms of time preference and negatively affect their self-management. This erratic behavior may be due to the release of stress hormones that impair the prefrontal cortex, a brain region associated with cognitive control, decision-making, and behavior regulation (Hirsh et al., 2008). Previous research has indicated that individuals with neurotic personalities tend to prefer smaller, immediate gratification rewards, which can have a negative impact on their ability to achieve long-term goals (Diekhof et al., 2012; Jimura et al., 2013). In contrast, patients with higher Conscientiousness scores are more inclined to delay gratification and show greater consistency in reward selection with respect to temporal preferences. Consequently, their adherence to diabetic self-management programs is higher (Axon et al., 2009). The findings of this study indicate that when designing interventions, greater emphasis should be placed on patients’ preferences to make intertemporal decisions. Measures should be implemented to help patients enhance their capacity to delay gratification, thereby improving their ability to self-manage.

The intertemporal decision preferences of patients with T2DM significantly impact their self-management behavior. These patients often face difficulties in adhering to self-management as they tend to choose immediate rewards over long-term benefits. To address this issue, a stepwise training approach is recommended. This approach would involve breaking down larger goals into smaller ones, gradually increasing patients’ confidence and self-efficacy (Gershman and Bhui, 2020). Patients with an immediate gratification preference can be provided with more immediate rewards to help them overcome difficulties. Previous studies suggest that individuals exhibit more patience when immediate rewards are more variable than delayed rewards (Elliott et al., 2023). Thus, a strategy that keeps delayed rewards relatively constant while imposing greater variability on immediate rewards may help encourage patients to be more patient. Secondly, most of the chronic disease governance prevention and control processes in China are developed in the context of consistent intertemporal decision-making, and existing policies often ignore individual differences, making it difficult for some patients to benefit from them (Lv and Deng, 2016). Therefore, in the prevention and control of chronic diseases, individualized interventions, and improved health promotion programs should be implemented by using intertemporal decision-making preferences as intervention targets, while more differentiated policy measures should be developed. In addition, Episodic future thinking has been shown to be an effective way to reduce delayed gratification preferences by actively imagining future events and reducing delayed discounting. This may be a promising avenue for future research (Stein et al., 2016).

This study provides valuable insights for improving self-management interventions for patients with T2DM. Healthcare providers can use positively-driven education to raise awareness among patients with an immediate gratification preference about the importance of self-management for managing their condition. However, this study has some limitations. First, it is a cross-sectional study and can determine only the correlation between variables; causal relationships cannot be ascertained. Secondly, the Cronbach’s alpha values for some dimensions of the self-management behavior scale we used are less than 0.7, which may have impacted the internal consistency of the scale. Consequently, we should exercise appropriate caution in interpreting the findings of this study. To rectify this issue, future research should consider using modified scales or other measures with higher internal consistency. In addition, the present study used a monetary discounting task to assess delay discounting, which may have some limitations in assessing intertemporal decision-making preferences in the context of diabetes management. Therefore, future studies should consider using more specific intertemporal decision-making tasks that specifically target diabetes management, including decisions about diet and exercise behaviors. Such tasks would allow for a more accurate assessment of the relationship between delayed discounting and self-management behaviors in people with diabetes and provide more effective interventions to improve self-management skills.

## 5. Conclusion

This study offers novel insights into the self-management capabilities of patients diagnosed with T2DM. The research finding emphasize the critical role of intertemporal decision-making in self-management among T2DM patients with traits of neuroticism and conscientiousness. As a result, interventions aimed at modifying intertemporal decision-making preferences have the potential to enhance self-management behaviors and improve health outcomes for patients with T2DM.

## Data availability statement

The original contributions presented in the study are included in the article/Supplementary material, further inquiries can be directed to the corresponding author.

## Ethics statement

The studies involving human participants were reviewed and approved by by the Ethics Committee of Jinzhou Medical University of Medical Sciences. The patients/participants provided their written informed consent to participate in this study.

## Author contributions

LD, JX, and SL conceived and designed the study. QF conducted the questionnaire collection. LD analyzed the data and wrote the manuscript. All authors read and approved the manuscript.

## Funding

This study was supported by the Natural Science Foundation (Project Number: 72174183) for Research on the Construction and Mode of 5G+ “Three Early Precautions” Health Management System.

## Conflict of interest

The authors declare that the research was conducted in the absence of any commercial or financial relationships that could be construed as a potential conflict of interest.

## Publisher’s note

All claims expressed in this article are solely those of the authors and do not necessarily represent those of their affiliated organizations, or those of the publisher, the editors and the reviewers. Any product that may be evaluated in this article, or claim that may be made by its manufacturer, is not guaranteed or endorsed by the publisher.

## Supplementary material

The Supplementary material for this article can be found online at: https://www.frontiersin.org/articles/10.3389/fpsyg.2023.1210691/full#supplementary-material
